# Production and statistical optimization of cholesterol-oxidase generated by *Streptomyces* sp. AN strain

**DOI:** 10.1186/s43141-022-00433-1

**Published:** 2022-11-10

**Authors:** Amany A. Alam, Doaa A. Goda, Nadia A. Soliman, Dina I. Abdel-Meguid, Ebaa E. El-Sharouny, Soraya A. Sabry

**Affiliations:** 1 Botany and Microbiology Department, Faculty of Science, Alexandria, Egypt; 2 Bioprocess Development Department, Genetic Engineering and Biotechnology Research Institute (GEBRI), City of Scientific Research and Technological Applications (SRTA-City),New Borg El-Arab City, Universities and Research Institutes Zone, P.O. 21934, Alexandria, Egypt

**Keywords:** Cholesterol oxidase, *Streptomyces* sp. AN, Experimental design, Process, Optimization

## Abstract

**Background:**

Cholesterol oxidases (CHOs) have attracted enormous attention because of their wide biotechnological potential. The present study explores the production of CHOs by *Streptomyces* sp. AN. Evaluation of culture conditions affecting enzyme production, medium optimization and released metabolite characteristics were also investigated.

**Results:**

The current work reports the isolation of 37 colonies (bacteria/actinobacteria) with different morphotypes from different soil/water samples. The isolate-coded AN was selected for its high potency for CHO production. Morphological characteristics and the obtained partial sequence of *16srRNA* of AN showed 99.38% identity to *Streptomyces* sp. strain P12–37. Factors affecting CHO production were evaluated using Plackett-Burman (PB) and Box-Behnken (BB) statistical designs to find out the optimum level of the most effective variables, namely, pH, starch, NH_4_NO_3_ and FeSO_4_.7H_2_O with a predicted activity of 6.56 U/mL. According to this optimization, the following medium composition was considered to be optimum (g/L): cholesterol 1, starch 6, MgSO_4_.7H_2_O 0.1, CaCl_2_ 0.01, FeSO_4_.7H_2_O 0.1, NH_4_NO_3_ 23.97, yeast extract (YE) 0.2, K_2_HPO_4_ 0.01, KH_2_PO_4_ 0.1, NaCl 0.01, Tween 20 0.01, pH 6.36 and incubation temperature (30 °C) for 9 days. Spectophotometric analysis for released metabolites against cholesterol (standard) via Fourier-transform infrared spectroscopy (FTIR) and differential scanning calorimetry (DSC) was carried out. FTIR spectrum showed the appearance of new absorption peaks at 1644 and 1725cm^−1^; this confirmed the presence of the Keto group (C=O) stretch bond. Besides, fermentation caused changes in thermal properties such as melting temperature peak (99.26; 148.77 °C), heat flow (− 8; − 3.6 Mw/mg), capacity (− 924.69; − 209.77 mJ) and heat enthalpy (− 385.29; 69.83 J/g) by comparison to the standard cholesterol as recognized through DSC thermogram. These changes are attributed to the action of the CHO enzyme and the release of keto derivatives of cholesterol with different properties.

**Conclusion:**

*Streptomyces* sp. AN was endowed with the capability to produce CHO. Enzyme maximization was followed using a statistical experimental approach, leading to a 2.6-fold increase in the overall activity compared to the basal condition. CHO catalyzed the oxidation of cholesterol; this was verified by the appearance of a new keto group (C=O) peak at 1644 and 1725 cm^−1^ observed by FTIR spectroscopic analysis. Also, DSC thermogram demonstrates the alteration of cholesterol triggered by CHO.

## Background

CHO is the enzyme that catalyzes the oxidation of cholesterol to cholestenone (cholest-4-en-3-one), with the reduction of an oxygen molecule to H_2_O_2_ (hydrogen peroxide) [1]. In recent times, microbial CHO has received great attention due to its wider use; CHO offers a broader range of industrial uses than clinical ones. The enzyme is used to analyse steroid levels in dietary samples. CHO is also used as a biosensor to measure serum cholesterol concentrations, which is crucial for diagnosing cardiovascular disease, atherosclerosis and other lipid disorders. CHO has also been implicated in the manifestation of viral diseases HIV, bacterial diseases (tuberculosis) and Alzheimer’s disease [2]. CHO exhibits anticancer properties in vitro when tested on rhabdomyosarcoma and breast cancer cell lines. It also possesses anticancer properties in an Ehrlich solid tumour model in vivo [3]. It has considerable insecticidal activity against the larvae of the *Anthonomus grandis* boll weevil, which decreases cotton yield [4]. In addition, for pimaricin (natamycin) production, *Streptomyces natalensis* CHO was utilized [5]. Significant attention has been received by CHO due to its wider use for the detection of cholesterol in food and blood samples, which has direct implications in lipid disorders including coronary heart diseases and atherosclerosis. Additionally, CHO is used in the production of steroids. Different bacteria have been shown to be involved in cholesterol degradation, while *Actinomycetes* are said to be the main group of organisms that degrade cholesterol.

Various bacterial species have been implicated in cholesterol biological degradation via a functionalized favin adenine dinucleotide-containing CHO that oxidizes cholesterol and creates 4-cholesten-3-one, while converting oxygen to hydrogen peroxide [6, 7]. The degradation of cholesterol by *Mycobacterium*, *Rhodococcus*, *Brevibacterium*, *Streptomyces* and some other gram-positive (G+) as well as gram-negative (G−) genera including *Comamonas*, *Burkholderia*, *Pseudomonas* and *Chromobacterium* has been reported [8–12].

*Streptomycetes*, like the other *Actinobacteria*, are G+ with a high GC content. Over two-thirds of the clinically relevant enzymes and antibiotics of natural origin are produced by *Streptomycetes*. The most productive source of microorganisms for all kinds of bioactive metabolites, including those with agro-active properties, is thought to be the *Streptomycetes*. In fact, *Streptomyces* is the source of nearly 60% of the novel insecticides and herbicides reported between 1988 and 1992 [13]. *Actinobacteria* are gaining popularity due to their low toxicity, specificity and environmentally friendly nature. However, for the development of commercially accessible *Actinomycete*-based products with a long shelf life, novel species must be identified as well as the mode of action of these bioagents should be further explored.

Thus, the aim of the present study was to isolate cholesterol-degrading microorganisms from different Egyptian localities and identify biochemically and genetically the most potent cholesterol-degrading isolate. The aim extended to optimize the nutritional and environmental conditions of the selected isolate to reach the highest productivity of CHO. Moreover, the developed end product (substrate degraded) was monitored and simply characterized through FTIR and DSC.

## Methods

### Bacterial isolation

Samples were gathered from various locations in Egypt (El Nubaria, West Sinai Desert and New Borg El-Arab City). Three samples were taken from El Nubaria petroleum wells at a depth of 40–50 cm, while samples from the west Sinai desert were taken at a depth of 20–30 cm. Another sample was taken from a waste oil facility and a soap factory in the City of Borg El Arab. At a depth of 15–20 cm, agricultural soil was also taken from New Borg El-Arab City. Sea water samples were also taken from two other seashores (the North coast and Marsa Matrouh City). All samples were collected in sterile 50-mL Falcon tubes, coded and kept at 4 °C until needed. Luria-Bertani (LB), composed of starch 10 g, peptone 2 g, YE 4 g and agar 15 g dissolved in 1 L distilled water, was used for bacterial isolation after adjusting the pH at 7.0 ± 0.1 [14]. One gram of soil or 1 mL of liquid was mixed with 9 mL of sterile distilled water. The diluted samples were streaked into sterile LB-agar medium poured into Petri dishes and incubated at 30 °C for 48–72 h. Morphological different colonies were selected, purified and maintained on sterile LB slants, coded and stored at 4 °C with regular transfer at monthly intervals.

### Qualitative screening for CHO activity (plate staining assay)

Qualitative screening for CHO production was done by growing different isolates on a selective medium (SM) composed of K_2_HPO_4_ 0.250 g, MgSO_4_7H_2_O 0.250 g, NaCl 0.005 g, FeSO_4_.7H_2_O 0.0005 g and cholesterol 1.0 g (as a sole carbon source) dissolved in 1 L of distilled water [15]. Several isolates were streaked on agar plates and incubated in an incubator at 30 °C for 7 days. Bacterial growth was used to assess bacteria’s ability to consume cholesterol and generate CHO. Filter paper discs were soaked in potassium phosphate buffer (pH 7.0) containing 0.5% cholesterol, 6% phenol, 1.7% 4-aminoantipyrine (4-AAP), and 3 U/mL of horse radish peroxidase. The soaked discs were then placed above the grown colonies and incubated at room temperature for another 24 h. The creation of red colour quinoneimine dye indicates CHO activity [16].

### Estimation of cholesterol degradation percentage

Monitoring of cholesterol degradation was carried out using the Bio Med-Cholesterol-LS kit (enzymatic colourimetric method) according to the manufacturer’s instructions. This used kit contains cholesterol standard reagent coded R1 with a concentration of 200 mg/dL and R2 reagent composed of Good’s buffer, peroxidase, 4-AAP and phenol derivatives. The reaction was carried out by mixing 10 μL sample (cell-free supernatant) with 1000 μL of R2 reagent. The reaction was incubated for 5 min at 37 °C and then read at 520 nm against blank which contains media free of inoculum. The standard was prepared in the same way using 10 μL of R1. Finally, the cholesterol degradation was calculated based on the following equation:

$$\textrm{Cholesterol}\ \left[\textrm{mg}/\textrm{dL}\right]=\textrm{Absorbance}\ \textrm{of}\ \textrm{test}/\textrm{Absorbance}\ \textrm{of}\ \textrm{standard}\times \textrm{Conc}.\textrm{of}\ \textrm{standard}\ \left[\textrm{mg}/\textrm{dL}\right]$$

### Quantitative determination of CHO activity

Wali and others employed the formation of hydrogen peroxide during the oxidation process of cholesterol to measure the activity of CHO [17]. A 100 μL of the enzyme was combined with 900 μL of the assay substrate, which included 87 mM potassium phosphate buffer, 0.89 mM cholesterol, 64 mM sodium cholate, 1.4 mM 4-aminoantipyrine, 21 mM phenol, 0.34% Tween 80 and 5 U/mL horse radish peroxidase. The reaction mixture was incubated for 5 min at 37 °C, and the generation of quinoneimine dye was monitored by measuring the absorbance at *λ*_520_. The activity of the enzyme was calculated according to the following formula:

$$\textrm{Unit}/\textrm{mLl}=\Delta \textrm{OD}/\min\ \left(\Delta \textrm{OD}\ \textrm{test}-\Delta \textrm{OD}\ \textrm{blank}\right)\ 13.78\times \textrm{Vs}\times \textrm{Vt}\times \textrm{df}$$

where Vt is the test’s total volume (1 mL), 13.78 is the quinoneimine dye’s millimolar extinction coefficient, df is the dilution factor, and Vs is the enzyme volume (100 μL) utilized in the experiment. Under the circumstances given above, one unit produces one micromole of hydrogen peroxide (half a micromole of quinoneimine dye) each minute.

### Morphological and biochemical characterization of the selected isolate

For the morphological characterization of the chosen isolate, a phase-contrast microscope (PCM) (AXIOSTA R-plus, ZEISS) was utilized. Scanning electron microscopy (SEM) was performed at 20 kV in the Centre Laboratory, City of Scientific Research and Technological Applications, using a JSM 5300 (JEOL, USA). The plate assay method was used for qualitative screening of the chosen isolate towards some distinct enzymes. Instead of starch, 0.2% of the equivalent substrate was added to the isolation medium to make agar plates. The clear zones were visualized using enzyme-specific methods. The substrates for the plate assay were carboxymethyl cellulose (CMC), starch, agarose, skim milk and tributyrin for cellulase, amylase, agarase, protease and lipase, respectively.

The production of cellulase was visualized by flooding the plates with congo red solution (0.1%), incubating for 30 min, removing the dye, and then fixing the colour with 2 M NaCl solution for 20 min. The presence of a pale orange zone around the colonies indicated a good positive result, while the rest of the plate was stained pink colour [18]. The production of agarase and amylase was visualized by flooding the plates with iodine-potassium iodide solution [19–21]; this reagent gives a translucent halo region around positive (agarase or amylase) colonies while the undegraded substrate(s) appeared in black-blue colour. Protease synthesis was determined by the formation of a clear zone around the colony [22]. Finally, for lipase, Arabic gum (1%) was dissolved in distilled H_2_O with gentle heating, and 0.2% tributyrin was added and emulsified well using Ultraturrax T25 blinder for 10 min, mixed with the medium and autoclaved, then poured in Petri dishes and inoculated with the tested isolate. The presence of a clear halo surrounding the colonies implies lipase/esterase synthesis [23].

### Identification using molecular techniques

*16S rRNA* gene sequencing was used to identify the most promising isolate possessing cholesterol-degrading activity. A genomic DNA was performed according to Kumar et al. [24]. The *16S rRNA* gene was amplified by polymerase chain reaction (PCR) using primers designed to amplify the full length (1500bp) of the *16S rRNA* gene according to the *Escherichia coli* (*E. coli*) genomic DNA sequence. A PCR reaction was completed, then a fraction of the PCR was evaluated on a 1% agarose gel according to the method published by Sambrook et al. [25], and the leftover mixture was purified using QIAquick PCR purification reagent (Qiagen Kit).

Based on the enzymatic chain terminator technique described by Sanger et al. [26], the DNA sequence was acquired using a 3130 X DNA Sequencer (Genetic Analyzer, Applied Biosystems, Hitachi, Japan). Using the nucleotide blast tool (BlASTn) [27], a nucleotide homology search was performed against *16s rRNA* sequences available in the database. Multiple sequence alignment and molecular phylogeny were performed using the MEGA software version 11 [28]. This alignment was used to create a neighbour joining (NJ) tree and then a maximum parsimony (MP) tree using bootstrapping [28].

### Optimization of culture condition using Plackett-Burman design (PBD)

Focusing on improving culture conditions, a progressive statistical-mathematical strategy was used to enhance the process of producing extracellular cholesterol-degrading enzymes from *Streptomyces* sp. AN. The first was a PBD-based screening of physicochemical variables. The second was BBD to optimize the most important factors that influence the enzyme production process.

The current study used an empirical design developed by Plakett and Burman, [29] to clarify the independent factors that would have a substantial impact on the performance of CHO production. In this study, a fractional factorial design of PB was used to determine whether twelve independent variables, namely NaCl, starch, YE, NH_4_NO_3_, Tween 20, K_2_HPO_4_, KH_2_PO_4_, incubation temperature, pH, MgSO_4_.7H_2_O, CaCl_2_ and FeSO_4_.7H_2_O, had any significant linear effect on extracellular CHO activity.

In the factorial design, the JMB software generated twelve test cases, as each factor was donated into two coded levels set to − 1, the low level, and +1, the high level. To depict the anticipated linear effect imposed by the tested independent variables on the process outcome in terms of CHO activity, an ordered polynomial equation was established.

$$Y=\beta 0+\sum \beta ixi$$

where *Y* represents the response (CHO activity), *β*0 represents the model’s intercept, *βi* represents the tested independent variable and *xi* represents the tested independent variable’s estimate. All of the experiments were carried out in 250-mL Erlenmeyer flasks with a 50-mL working volume and agitation rate (200 rpm), and all trials were carried out three times. The Pareto diagram is the best way to express the PB results since it shows the absolute relative significance of variables regardless of their nature [29].

For the next optimization phase, a conformational step should be performed. Variables with negative effect values were fixed to their − 1 coded values, while those with positive effect values were fixed to their + 1 coded values. The goal of this stage is to verify the PBD results and build the basic formula for the next optimization step.

### Response surface method (RSM) to optimize CHO production

Each independent variable that had a substantial impact on extracellular CHO yield as determined by PBD was subjected to RSM using BBD [30–32]. Where the most significant four variables were selected to determine their optimal level with respect to CHO activity (U/mL) as a response, a second-order polynomial function was fitted to correlate the relationship between the independent variables and the response. The applied design matrix consists of twenty-seven trials with three runs at the centre point and three levels donated by (− 1, 0, 1) for the selected variables (pH, starch, NH_4_NO_3_ and FeSO_4_.7H_2_O), where the design was used to determine the optimal level of the significant factors for CHO production. Through the following second-order polynomial equation for the four variables, BBD concludes all potential interactions among the specified independent factors that would affect the outcome:

$$\textrm{Y}={\upbeta}_0+{\upbeta}_1\left({\textrm{X}}_1\right)+{\upbeta}_2\left({\textrm{X}}_2\right)+{\upbeta}_3\ \left({\textrm{X}}_3\right)+{\upbeta}_4\left({\textrm{X}}_4\right)+{\upbeta}_{12}\left({\textrm{X}}_1{\textrm{X}}_2\right)+{\upbeta}_{13}\left({\textrm{X}}_1{\textrm{X}}_3\right)+{\upbeta}_{14}\left({\textrm{X}}_1{\textrm{X}}_4\right)+{\upbeta}_{23}\left({\textrm{X}}_2{\textrm{X}}_3\right)+{\upbeta}_{24}\left({\textrm{X}}_2{\textrm{X}}_4\right)+{\upbeta}_{34}\left({\textrm{X}}_3{\textrm{X}}_4\right)\kern0.5em +{\upbeta}_{11}{\left({\textrm{X}}_1\right)}^2+{\upbeta}_{22}{\left({\textrm{X}}_2\right)}^2+{\upbeta}_{33}{\left({\textrm{X}}_3\right)}^2+{\upbeta}_{44}{\left({\textrm{X}}_4\right)}^2$$

where *Y* is the predicted response; *β*_0_ is the constant; *β*_1_, *β*_2_, *β*_3_ and *β*_4_ are the linear coefficients; *β*_12_, *β*_13_, *β*_14_, *β*_23_, *β*_24_ and *β*_34_ are the cross product coefficients; and *β*_11_, *β*_22_, *β*_33_ and *β*_44_ are quadratic coefficients.

### Data analysis

Multiple linear regressions were performed on the CHO production data using the JMP tool to estimate the *t* values, *p* values and confidence levels, with the *p* values expressed as a percentage. The *Student t test* was used to evaluate the significance level (*p value*). The *t test* for any individual effect allows for an assessment of the likelihood of discovering the observed effect by chance. It will be acceptable if the probability of the variable under test is modest enough. The confidence level is a percentage representation of the *p value*. The JMP software was used to calculate the ideal value of the activity. Three-dimensional and contour plots were prepared by the STATISTI CA 7.0 software in order to display the simultaneous effects of the four most important independent factors on each response.

### Model validation

The ideal settings discovered through optimization trials were tested experimentally and compared to the model’s data.

### Cholesterol metabolites analysis using FTIR

The cholesterol metabolites formed during the breakdown process were identified using FTIR analysis against the intact substrate (undegraded). The FTIR (Shimadzu FTIR-84 00 S, Japan) is linked to a PC, and the data was analysed with the IR Solution programme, version 1.21. The scan range for each sample was 4000 to 5000 cm^−1^, with a resolution of 1 cm^−1^.

### DSC analysis

After fermentation, lyophilized samples were exposed to DSC-60A to determine their pyrolysis pattern and compared to the control (un-degraded cholesterol). The experiment was carried out in a nitrogen atmosphere with a 10 °C min^−1^ heating rate and a 30 mL min^−1^ flow rate. The thermogram was taken at temperatures ranging from 25 to 350 °C. Temperature vs. heat flow was displayed on the graph.

## Results

### Isolation and screening for CHO

Among 60 obtained isolates, 37 different morphotypes (shapes/colour) were selected and subjected to the colony staining method to test for cholesterol degradation and CHO production. The strains were first screened for qualitative estimation of CHO activity on plates containing 0.1% cholesterol as the only carbon source. Five isolates showed an ability to grow in the presence of cholesterol as a carbon source. Based on the intensity of the red colour appearance, the isolates can be ordered as follows, AN > PI > MR1 > AIR2. These isolates showed the same order based on cholesterol degradation (98.02, 74.6, 41.9 and 9.6%) according to the kit assay result (full data not shown). Quantitative detection of CHO by the examined isolates depicts that all were able to produce CHO (2.54, 1.32, 0.92 and 0.6 U/mL) after 9 days of incubation. Therefore, stain AN was selected for further study, because it was recognized as the most active in relation to colour intensity (plate assay), the highest titer of CHO activity (2.5 U/ml) and the highest degradation % of cholesterol (98%) through kit assay.

### Enzymatic profile of the selected isolate AN

Luria-Bertani media (LB) agar plates with 5 different substrates adjusted at pH 7.0 and incubated at 30 °C were prepared. By using the suitable indicator for each enzyme, it was observed that AN isolate was able to produce lipase (tributryrin-substrate) and CHO while being negative for amylase, protease and agarase.

### Morphological and molecular features of AN

**Fig. 1:**
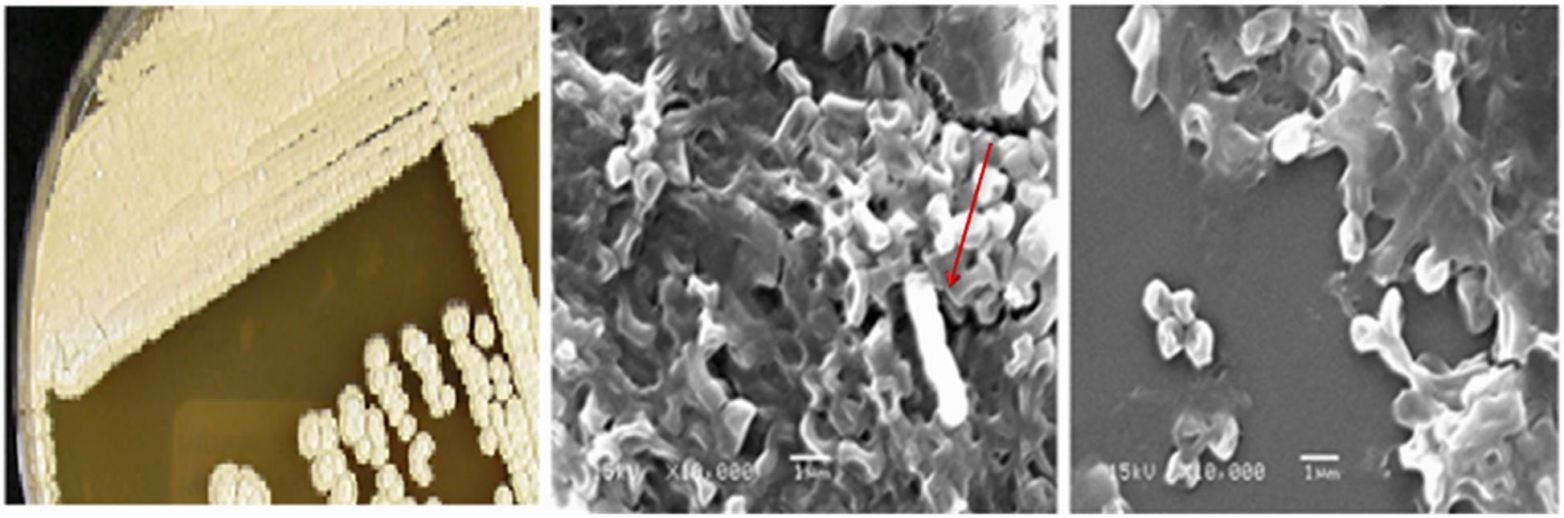
Colony morphology of AN-coded isolate on LB agar plate incubated at 30 °C for 3 days (left) and SEM micrographs (middle and right). The red arrow pointed to the spore structure-like sporangium

**Fig. 2:**
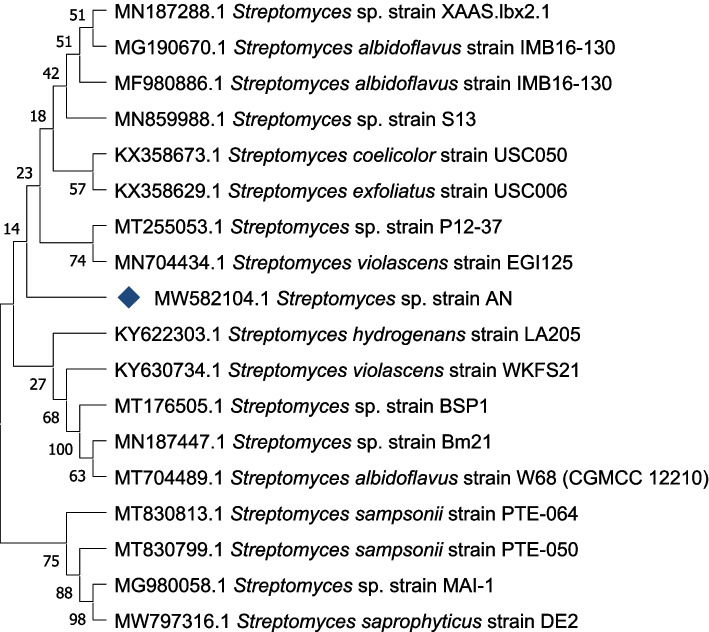
A phylogenetic tree, based on the 16S *rRNA* gene sequence comparison showing the position of *Streptomyces* sp. strain AN, and its closest relatives

AN was G+ characterized by the presence of creamy-brown mycelia on LB agar plates. Under SEM, mycelia formed a spore structure-like sporangium (Fig. 1). The partial sequencing of AN (971 bps) was submitted to the BLAST in order to find homologies with other relevant *16S rRNA* sequences. This partial sequence of AN-coded isolate showed 99.38% identity to *Streptomyces* sp. strain P12-37 (AC: MT255053.1). Subsequently, the investigated AN strain was deposited in GenBank under accession number (AC: MW582104.1) and designated *Streptomyces* sp. strain AN. The phylogeny of the AN strain (Fig. 2) explained that it localized in the cluster included p12–37 and EG1125 strains but closer to *S. violascens* strain EG1125 (AC: MN704434).

### Statistical optimization for CHO production by multi-factorial experiment

PBD design (the first approach) was applied to evaluate the relative significance of cultivation variables affecting the production of CHO by *Streptomyces* sp. AN.

In attendance, twelve different variables including nutritional factors such as carbon source (starch, Tween 20), nitrogen source (YE; NH_4_NO_3_) and salts (FeSO_4_.7H_2_O, K_2_HPO_4_, KH_2_PO_4_, NaCl, CaCl_2_, MgSO_4_.7H_2_O) and physical factors such as pH and temperature were evaluated. The averages of the CHO activities (response U/mL) showed a wide variation from 2.54 to 4.41 U/mL (Table 1).

**Table 1:** Randomized PB matrix designed for evaluating the factors influencing the CHO production by *Streptomyces* sp. strain AN

Trials	Variables												CHO (U/ml)		
	X1	X2	X3	X4	X5	X6	X7	X8	X9	X10	X11	X12	Actual	Predicted	Residual
1	− 1	1	1	1	1	− 1	1	− 1	1	1	1	− 1	4.53	4.58	− 0.054
2	1	− 1	− 1	− 1	1	− 1	− 1	− 1	− 1	1	1	− 1	3.48	3.50	− 0.01
3	− 1	− 1	− 1	− 1	1	1	1	1	1	− 1	− 1	− 1	3.33	3.28	0.064
4	1	1	− 1	− 1	1	1	1	− 1	1	− 1	1	1	3.48	3.55	− 0.064
5	1	− 1	1	1	1	1	− 1	1	1	1	− 1	1	4.09	4.05	0.054
6	− 1	− 1	1	1	1	1	− 1	− 1	− 1	− 1	1	1	4.11	4.052	0.064
7	1	− 1	− 1	1	− 1	1	1	− 1	− 1	1	− 1	− 1	3.60	3.66	− 0.054
8	1	1	− 1	1	− 1	− 1	− 1	− 1	1	− 1	− 1	1	3.58	3.59	− 0.011
9	1	1	1	1	1	− 1	1	1	− 1	− 1	− 1	− 1	2.58	2.58	− 0.001
10	1	− 1	1	− 1	− 1	− 1	− 1	1	1	− 1	1	− 1	3.79	3.74	0.054
11	1	1	1	− 1	− 1	1	1	1	− 1	1	1	1	3.83	3.83	0.001
12	− 1	− 1	1	− 1	− 1	− 1	1	− 1	− 1	− 1	− 1	1	3.25	3.32	− 0.064
13	− 1	1	− 1	1	− 1	1	− 1	1	− 1	− 1	1	− 1	4.12	4.11	0.011
14	− 1	1	− 1	− 1	1	− 1	− 1	1	− 1	1	− 1	1	2.53	2.52	0.011
15	− 1	1	1	− 1	− 1	1	− 1	− 1	1	1	− 1	− 1	4.41	4.42	− 0.001
16	− 1	− 1	− 1	1	− 1	− 1	1	1	1	1	1	1	3.62	3.63	0.001
Variables		Code	Coded level and actual level												
			− 1	1											
Starch (g%)		X1	0.2	2											
MgSO_4_.7H_2_O (g%)		X2	0.01	0.1											
CaCl (g%)		X3	0.01	0.1											
FeSO_4_.7H_2_O (g%)		X4	0.01	0.1											
NH_4_NO_3_ (g%)		X5	2.0	20											
YE (g%)		X6	0.2	2											
K_2_HPO_4_ (g%)		X7	0.01	0.1											
KH_2_PO_4_ (g%)		X8	0.01	0.1											
NaCL (g%)		X9	0.01	0.1											
pH (value)		X10	5	8											
Tween 20 (g%)		X11	0.01	0.1											
T (°C)		X12	30	37											

Based on the regression analysis shown in Table 2; the regression coefficient of the variables, namely, starch, FeSO_4._7H_2_O, NH_4_NO_3_, MgSO_4_.7H_2_O and KH_2_PO_4_, showed a positive effect on CHO activity where cultivation temperature, pH, Tween 20, YE, K_2_HPO_4_ and NaCl contributed negatively. The 12 variables were analysed using a linear multiple regression analysis method, and the % confidence level was calculated [confidence level % = (1 − *p* value) × 100]. Also, the main effect was calculated basically as a difference between the average measurements of each variable made at a high level (+ 1) and a low level (− 1) (Table 2).

**Table 2:** Statistical analysis of PB showing *coefficients* and *t* and *p* values for variables affecting the CHO production by *Streptomyces* sp. strain AN

Term	Coefficients	Main effect	Standard error	*t* Stat	*p* value	Confidence level (%)
Intercept	3.651436		0.0076	150.4767	6.47134E−07	
Starch	0.268015	0.53603	0.0076	11.04499	0.001589662	99.84103
MgSO_4_.7H_2_O	0.03652	0.07304	0.0076	1.505014	0.229383869	77.06161
CaCl_2_	− 0.01257	− 0.02514	0.0076	− 0.51812	0.64016232	35.98377
FeSO_4_.7H_2_O	0.127123	0.254246	0.0076	5.238763	0.013538168	98.64618
NH_4_NO_3_	0.092798	0.185596	0.0076	3.824215	0.031483379	96.85166
YE	− 0.11515	− 0.2303	0.0076	− 4.74532	0.017752588	98.22474
K_2_HPO_4_	− 0.06885	− 0.1377	0.0076	− 2.83732	0.065791541	93.42085
KH_2_PO_4_	0.004191	0.008382	0.0076	0.172706	0.873876628	12.61234
NaCl	− 0.00818	− 0.01636	0.0076	− 0.33719	0.758187184	24.18128
pH	− 0.37179	− 0.74358	0.0076	− 15.3215	0.000603871	99.93961
Tween 20	− 0.16065	− 0.3213	0.0076	− 6.62042	0.007018534	99.29815
T (°C)	− 0.12233	− 0.24466	0.0076	− 5.04138	0.015048308	98.49517
ANOVA analysis	*df*	*SS*	*MS*	*F*	*Significance F*	
Regression	12	0.463234	0.038603	41.77511	0.005287	
Residual	3	0.002772	0.000924			
Total	15	0.466006				
Multiple *R*	0.997021					
*R*^2^ square	0.994051					
Adjusted *R*^2^	0.970256					

**Fig. 3:**
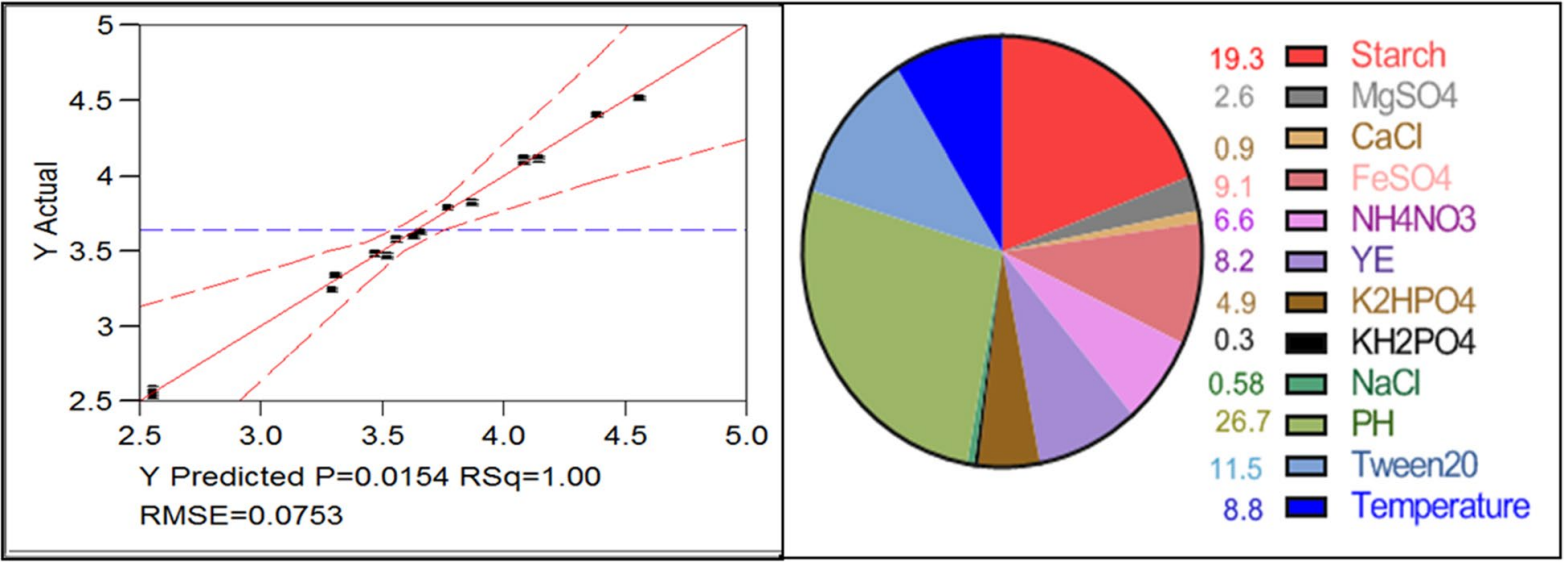
Actual by predicted correlation plot in PB experiment (left) and a Pareto diagram for PB analysis, downsize the effect of each variable (%) on CHO production (right)

The ranking of factor estimates is shown in a Pareto diagram (Fig. 3), where starch has the highest effect (19%) while NaCl has the lowest (0.58%). The *p* value from the ANOVA analysis for each response was determined to analyse the relationship between the variables.

The analysis of variance using the ANOVA test gives *p* = 0.0052, indicating a statistically significant relationship between the variables at a 99.99% confidence level. The *R-squared* statistic indicates that the model as fitted explains 0.99 of the variability in CHO activity. At the model level, the correlation measure for the estimation of the regression equation is the multiple correlation coefficients *R* and the determination coefficients *R*^2^. The closer the value of *R* to 1, the better the correlation between the measured and the predicted values as shown in Fig. 3. The polynomial model describing the correlation between the 12 factors and the CHO activity could be presented as follows: *Y* = 3.651436 + 0.268015 *X*_1_+ 0.03652 *X*_2_ − 0.01257 *X*_3_ + 0.127123 *X*_4_ + 0.092798 *X*_5_ − 0.11515 *X*_6_ − 0.06885 *X*_7_ + 0.004191 *X*_8_ − 0.00818 *X*_9_ − 0.37179 *X*_10_ − 0.16065 *X*_11_ − 0.12233 *X*_12._

On the basis of the calculated *t* values and confidence levels (%), starch, pH, FeSO_4_.7H_2_O, temperature and NH_4_NO_3_ of confidence level ≥ 96% were found to be the most significant variables affecting CHO activity produced by *Streptomyces* sp. AN. According to these results, a medium of the following composition (g/L), cholesterol 1, starch 2, MgSO_4_ 0.1, CaCl_2_ 0.01, FeSO_4_.7H_2_O 0.1, NH_4_NO_3_ 20, YE 0.2, K_2_HPO_4_ 0.01, KH_2_PO_4_ 0.1, NaCl, 0.01, Tween 20 0.01 and pH 5, and incubation at 30 °C for 9 days under shaking (200rpm) was used as the basic medium for the next design.

BBD (the second approach) was applied in order to reach the optimum response region for CHO production; the significant independent variables {pH (*X*_1_), starch (*X*_2_), NH_4_NO_3_ (*X*_3_), FeSO_4._7H_4_O (*X*_4_)} were further explored at three levels based on the results obtained in PBD. Table 3 represents the levels of each tested variable in coded units, − 1 (low level), 0 (medium level) and 1 (high level). The four variables with 27 trials were analysed using the linear multiple regression analysis method, and the percentage of confidence level was calculated. The analysis of variance using the ANOVA test in the BB experiment was generated and summarized in Table 4, which gives *p* = 0.0123. Since the *p* value indicated in the ANOVA table is less than 0.05, it is concluded that there is a statistically significant relationship among the studied variables at a 95% confidence level (*p* = 0.05). The value of the determination coefficient *R*^2^ = 0.80 for CHO activity, being a measure of fit of the model, indicates that about 20% of the total variations are not explained by CHO activity.

**Table 3:** BB designed matrix for the selected 4 variables influencing the CHO production by *Streptomyces* sp. strain AN

Trial	Variables				CHO (U/mL)		
	X1	X2	X3	X4	Experimental	Predicted	Residual
1	0	0	0	0	6.398844	6.40523	− 0.00639
2	0	0	1	− 1	6.066768	6.161761	− 0.09499
3	− 1	− 1	0	0	5.830484	5.839797	− 0.00931
4	0	0	1	1	6.073154	6.185709	− 0.11255
5	0	0	− 1	1	5.986943	6.027654	− 0.04071
6	1	− 1	0	0	6.057189	6.226686	− 0.1695
7	0	0	− 1	− 1	6.220034	6.243184	− 0.02315
8	− 1	1	0	0	6.175332	6.141539	0.033793
9	1	1	0	0	6.232806	6.359197	− 0.12639
10	0	0	0	0	6.424388	6.40523	0.019158
11	0	1	1	0	6.26793	6.309439	− 0.04151
12	0	− 1	1	0	5.970977	5.990136	− 0.01916
13	0	1	− 1	0	6.204069	6.168946	0.035123
14	1	0	0	− 1	6.376493	6.345627	0.030866
15	− 1	0	0	1	5.932661	5.947562	− 0.0149
16	0	− 1	− 1	0	6.111471	6.053996	0.057475
17	− 1	0	0	− 1	6.223227	6.081669	0.141558
18	1	0	0	1	6.162559	6.288152	− 0.12559
19	0	− 1	0	− 1	5.734693	5.751456	− 0.01676
20	0	1	0	1	6.009294	5.872791	0.136502
21	0	1	0	− 1	6.501021	6.538539	− 0.03752
22	− 1	0	− 1	0	5.983749	6.133556	− 0.14981
23	0	0	0	0	6.392458	6.40523	− 0.01277
24	1	0	1	0	6.743692	6.474147	0.269545
25	1	0	− 1	0	6.347756	6.226686	0.121069
26	0	− 1	0	1	6.382879	6.225622	0.157257
27	− 1	0	1	0	5.961398	5.962729	− 0.00133
Variables	Code	Coded level and actual level					
		− 1	0	1			
pH	X1	5	6	7			
Starch (g/L)	X2	2	4	6			
NH_4_NO_3_ (g/L)	X3	20	25	30			
FeSO_4_.7H_2_O (g/L)	X4	0.1	0.15	0.2

**Table 4:** Statistical analysis of BB design showing *coefficients* and *t* and *p values* for significant variables affecting the CHO by *Streptomyces* sp. strain AN

Term	Coefficients	Standard error	*t* Stat	*p* value	Upper 95.0%
Intercept	6.40523	0.086412	74.12	2.42E−17	2.064964
X1&RS	0.151137	0.043206	3.5	0.0044	99.56026
X2&RS	0.108563	0.043206	2.51	0.0273	97.27277
X3&RS	0.019158	0.043206	0.44	0.6654	33.46475
X4&RS	− 0.0479	0.043206	− 1.11	0.2894	71.06497
X1*X2	− 0.04231	0.074835	− 0.57	0.5823	41.77478
X1*X3	0.104572	0.074835	1.4	0.1876	81.2398
X2*X3	0.051089	0.074835	0.68	0.5078	49.22296
X1*X4	0.019158	0.074835	0.26	0.8023	19.7719
X2*X4	− 0.28498	0.074835	− 3.81	0.0025	99.75072
X3*X4	0.059869	0.074835	0.8	0.4392	56.07516
X1*X1	− 0.09739	0.064809	− 1.5	0.1588	84.12301
X2*X2	− 0.16604	0.064809	− 2.56	0.0249	97.50889
X3*X3	− 0.10856	0.064809	− 1.68	0.1197	88.02517
X4*X4	− 0.14209	0.064809	− 2.19	0.0488	95.12073
ANOVA	*df*	*SS*	*MS*	*F*	Prob > F
Model	14	0.102921	0.007352	3.3459	0.0213
Error	12	0.026366	0.002197		
C. total	26	0.129287			
*R*^2^	0.796068				
*R*^2^ Adj	0.558148				
Root mean square error	0.046874				
Mean of response	1.934444				
Observations (or sum Wgts)	27				

**Fig. 4:**
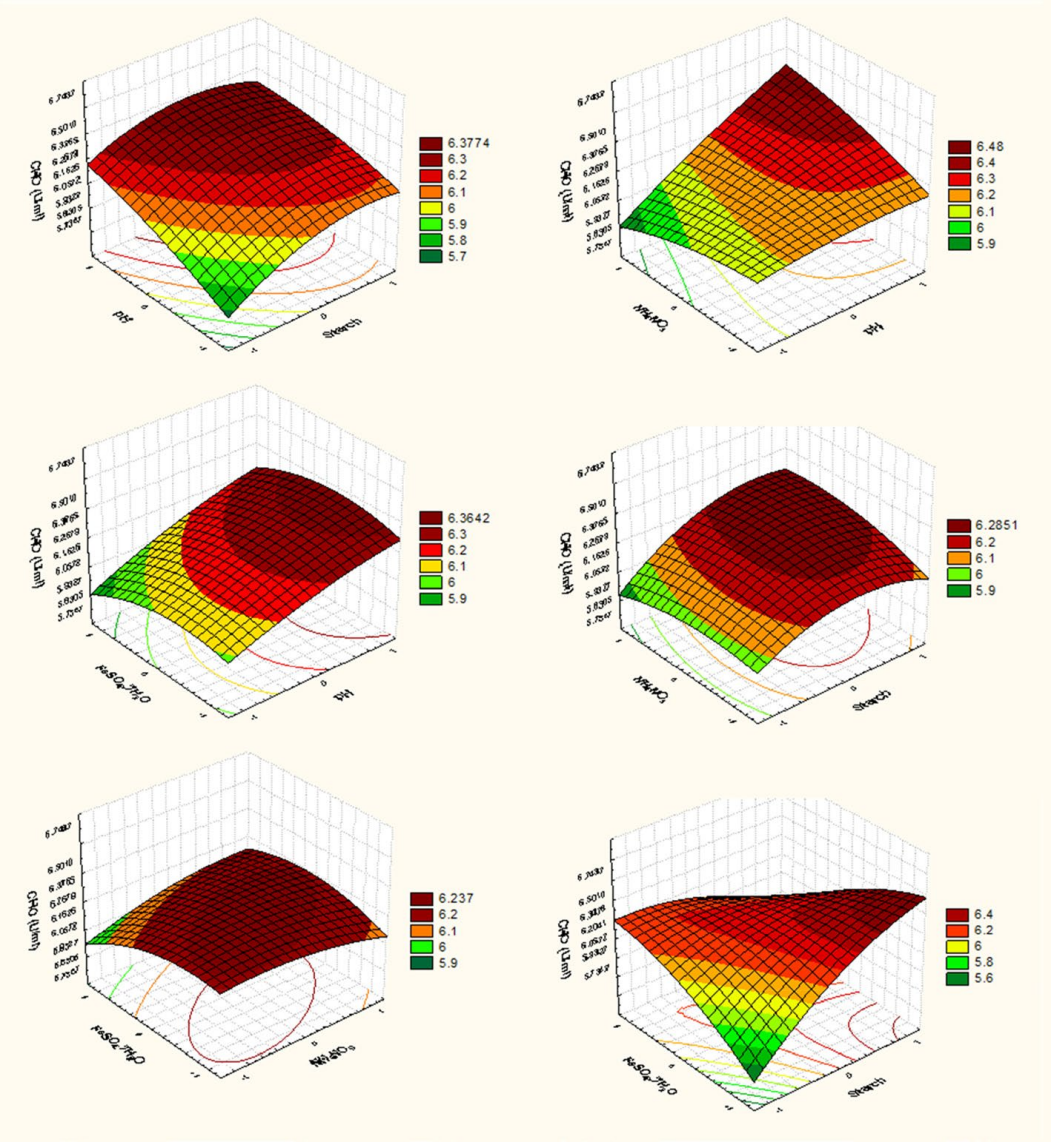
Three-dimensional surface and contour plots showing the relationships between the tested variables and the CHO produced by *Streptomyces* sp. AN

The multiple linear regression models describe the relationship between the enzyme activity and four studied variables, namely, pH, starch, NH_4_NO_3_ and FeSO_4._7H_2_O. Surface plots (Fig. 4) show that higher levels of CHO activity were attained by increasing the concentration of starch and pH while decreasing the iron level and using a concentration near to the low value of ammonium nitrate in the medium. Contour analysis (Fig. 4) was calculated for *Streptomyces* sp. AN CHO to detect the centre point which gives maximum CHO activity.

For predicting the optimal point, a second-order polynomial function was fitted to the experimental results (linear optimization algorithm) for CHO.

$$Y=6.40523+0.151137{X}_1+0.108563{\textrm{X}}_2+0.019158{X}_3-0.0479{X}_4-0.04231{\textrm{X}}_1{\textrm{X}}_2+104572{\textrm{X}}_1{\textrm{X}}_3+0.019158{\textrm{X}}_1{\textrm{X}}_4+0.051089{X}_2{X}_3-0.28498{X}_2{X}_4+0.059869{X}_3{X}_4-0.09739{X}_1{X}_1-0.16604{X}_2{X}_2-0.10856{X}_3{X}_3-0.14209{X}_4{X}_4$$

where *X*_1_, *X*_2_, *X*3 and *X*_4_ are pH, starch, NH_4_NO_3_ and FeSO_4_.7H_2_O, respectively.

**Fig. 5:**
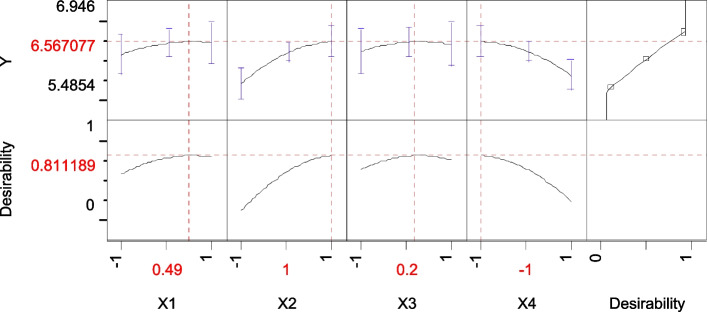
JMP desirability prediction profile showing the predicted optimal coded levels (0.4555, 1, 0.35166 and − 1) of studied four variables pH, starch, NH_4_NO_3_ and FeSO_4_.7H_2_O, respectively, to maximize the CHO by *Streptomyces* sp. AN

The optimal levels of the four components as obtained from the maximum point of the polynomial model were estimated using the *SOLVER* function of Microsoft Excel tools and *JMP*-program and found to be (g/L) starch 6.0, NH_4_NO_3_ 23.97 and FeSO47H2O 0.1 at pH 6.36, with a predicted activity of 6.56 U/mL (Fig. 5).

In order to determine the accuracy of the quadratic polynomial, a verification experiment was carried out under the predicted optimal condition as determined previously. To prove the accuracy of the model, the % accuracy was calculated from the following formula:

$$\textrm{Accuracy}\ \textrm{of}\ \textrm{the}\ \textrm{model}=\left\{Y\textrm{experiment}/Y\textrm{calculated}\right\}\times \textrm{x}100$$

The estimated activity of *Streptomyces* sp. AN CHO was 6.61 U/mL. This means that the calculated model accuracy was 99.2%. In this study, the combination of PB and BB designs was shown to be effective and reliable in selecting the statistically significant factors and finding the optimal concentration of each factor.

Based on the results obtained from the PB and BB designs, the expected medium composition for optimum CHO activity by *Streptomyces* sp. AN was (g/L) cholesterol 1, starch 6, MgSO_4_.7H_2_O 0.1, CaCl_2_ 0.01, FeSO_4_.7H_2_O 0.1, NH_4_NO_3_ 23..97, YE 0.2, K_2_HPO_4_ 0.01, KH_2_PO_4_ 0.1, NaCl 0.01, Tween 20, 0.01, pH 6.36 and incubation temperature 30 °C for 9 days.

Finally, the production of *Streptomyces* sp. AN CHO has been systematically improved by almost 2.6-folds during various experimental designs compared with the basal medium.

### Fourier-transform infrared (FTIR) and DSC spectroscopic analyses of released metabolites

**Fig. 6:**
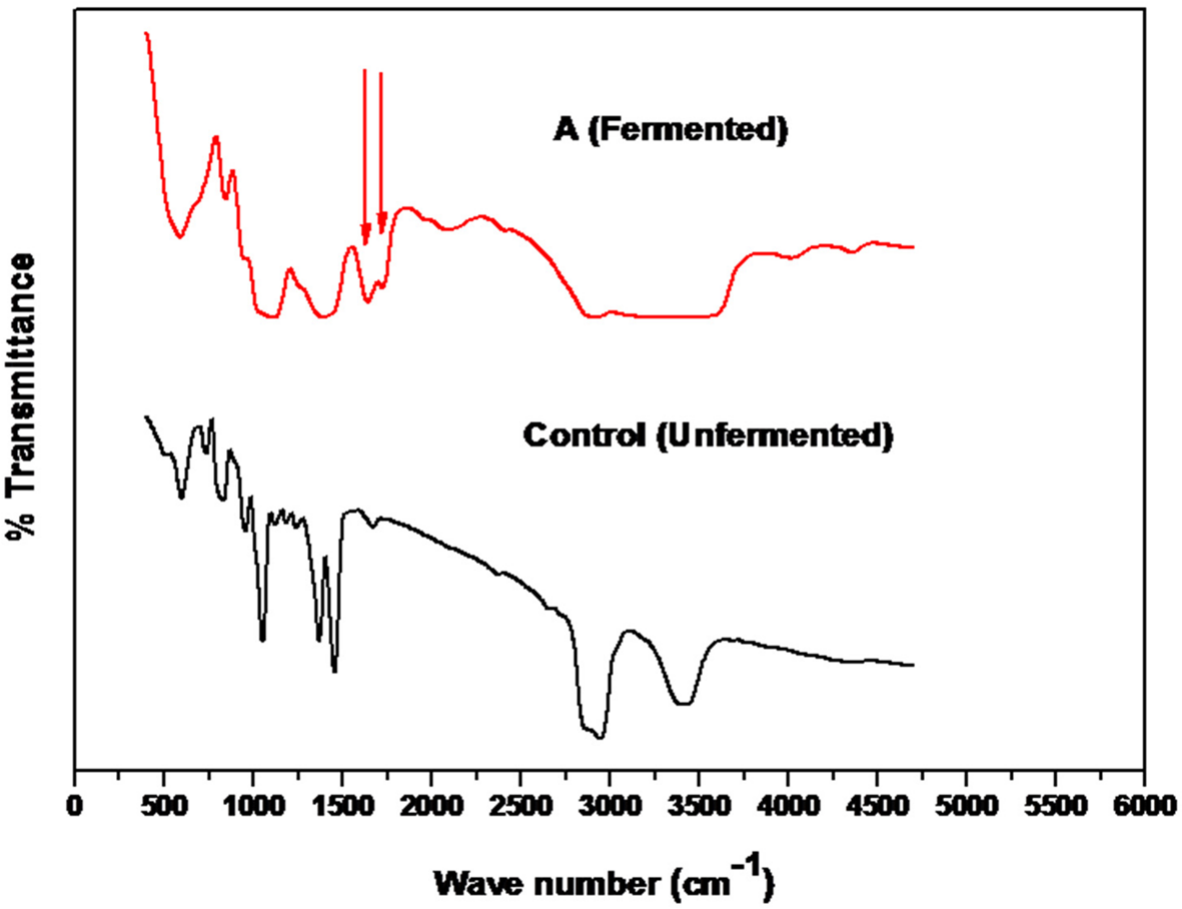
FTIR analyses of cholesterol degradation by *Streptomyces* sp. AN. FTIR pattern for cholesterol substrate powder, control (unfermented), and A (fermented) after the fermentation process. The arrow point to the presence of the Keto group C=O

Alterations related to the action of CHO produced by *Streptomyces* sp. AN were examined using FTIR spectra of cholesterol (substrate) powder samples before and after the fermentation course. Before the fermentation bioprocess, the FTIR spectrum of the substrate revealed the presence of absorption peaks at 3401, 2944, 1456, 1370, 1054, 955, 833 and 589.9 cm^−1^ (Fig. 6, control).

These typical absorption peaks and intensity were noticed to be changed after fermentation. The absorption maxima at 3401, 2944, 1456, 1370 and 1054 cm^−1^ in the control (unfermented cholesterol substrate) were considerably reduced by fermentation, and new distinctive absorption peaks at 1644 and 1725 cm^−1^ were developed in fermented samples (Fig. 6, A).

### Differential scanning calorimetry (DSC) analysis

**Fig. 7:**
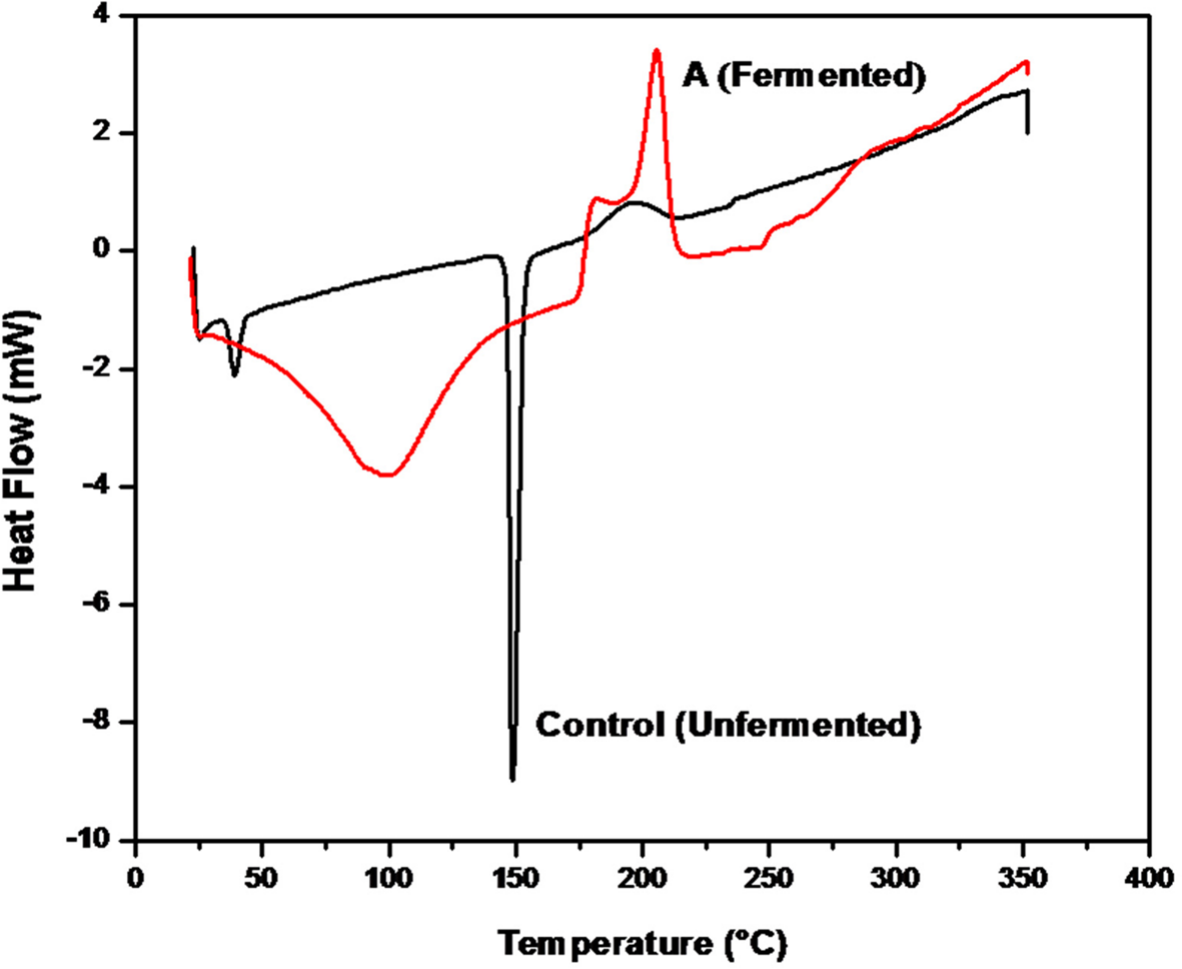
DSC analysis pattern for cholesterol substrate powder control (unfermented), and A (fermented) after the fermentation process under optimal conditions

The thermal characteristics of cholesterol substrate and released products after fermentation were estimated qualitatively and quantitatively using DSC analysis. The DSC-control thermogram (Fig. 7, control) revealed the normal endothermic transition of the cholesterol substrate powder, with melting temperatures peaking at 38.96, 148.77 and 196.24 °C and heat flow, capacity, and enthalpy (− 2 mW/mg, − 225.13 mJ and − 8.38 J/g; − 8 mW/mg, − 209.78 mJ and − 69.93 J/g; and 1 mW/mg, 49.73 mJ and 16.58 J/g, respectively). By comparing the DSC of the fermented product (Fig. 7, A) produced after optimization to the control-DSC, noticeable differences in the melting temperature peak (99.26 °C), heat flow (− 3.6 mW/mg), heat capacity (− 924.69 mJ) and heat enthalpy (− 385.29 J/g) were recognized.

## Discussion

Numerous scholars have noted that the formation of a red hue is caused by the formation of quinoneimine dye, indicating the development of CHO [16, 17, 33]. Similarly, the investigated bacterial strain *Streptomyces* sp. NA proved to produce CHO. In order to increase CHO production by *Streptomyces* sp. AN, a sequential optimization technique was adopted in two steps (PB and BB). El-Naggar et al. [34] investigated the effects of environmental and metabolic variables on *Streptomyces cavourensis* strain NEAE-42’s CHO. PB design offers a simple, quick screening process as well as statistically evaluating the significance of a large number of variables in one experiment, saving time and ensuring that each element has persuasive evidence. In this study, PB results showed a wide variation in CHO activity from 2.54 to 4.41 U/mL. According to the *p* values of the studied 12 variables, starch, pH, FeSO_4_.7H_2_O, temperature and NH_4_NO_3_ were found to be the most significant affecting CHO activity generated by *Streptomyces* sp. AN. Our results are significantly better than those of *S. niger* MTCC 4010 (0.27 U/mL), *S. fradiae* MTCC 4002 (0.32 U/mL), *S. olivaceus* MTCC 6820 (0.625 U/mL), *S. hygroscopicus* MTCC 4003 (0.472 U/mL), *S. annulatus* MTCC 6818 (0.355 U/mL) and *S. clavifer* MTCC 4150 (0.254 U/mL) [35].

In a previous study, some physical factors including the initial pH of the medium, cultivation temperature and shaking speed affecting the production of CHO by *Rhodococcus equi* were studied [36]. Also, medium pH, incubation temperature, inoculum size, inoculum age, fermentation period and shaking speed were studied for augmenting the CHO production by *S. olivaceus* MTCC 6820 [37].

For microbial growth, metabolic characteristics and metabolite production, the pH value of the cultural medium is critical. According to [38], changes in the pH of the culture medium have a considerable impact on the cells’ optimal physiological performance, the transfer of different nutrients via the cell membrane and cholesterol breakdown.

Many investigators believe that confidence levels of variables greater than 90% are suitable and acceptable when evaluating the empirical statistical model [39, 40] for bioprocess optimization. This current investigation’s variables showed a confidence per cent greater than 96.85, *p* value ≥ 0.031. Additionally, one of the PBD’s advantages is that it allows operators to rank the effect of different variables on the measured response regardless of the factor’s nature.

The medium components are improved by statistical approaches (RSM) in the second step of optimizing CHO production. The important contributing variables (pH, starch, NH_4_NO_3_ and FeSO_4_.7H_2_O) were further investigated at three levels: − 1, 0 and + 1 in order to approach the CHO’s optimum response area with activity. The findings of surface plots revealed that raising the starch concentration and pH value while lowering the iron and ammonium nitrate levels resulted in higher levels of CHO activity.

The *R*^2^ value for CHO activity in this design was 0.99, indicating a strong correlation between the actual and anticipated values. The experimentally verified optimal settings from the optimization experiment were compared to the model’s anticipated optimum. The estimated CHO activity was 6.61 U/mL, and the polynomial model predicted a value of 6.56 U/mL. This high level of accuracy (99.2%) indicates that the model was validated under ideal conditions. Furthermore, the enzyme activity in the improved medium was 2.6 times higher than in the baseline conditions. This demonstrated the importance and usage of the optimization process. Our findings are consistent with [39] in which the RSM is a commonly accepted advanced numerical method for optimizing experimental conditions and solving analysis problems in which a response is strongly impacted by many variables for the production of industrially important biological molecules.

After the fermentation process and degradation of cholesterol by excreted enzymes, primarily CHO produced by *Streptomyces*. sp. AN, caused changes in the existing functional groups of cholesterol. CHO reacted with the side chain of cholesterol, causing changes and modifications to the cholesterol’s existing functional groups. The new distinctive absorption peaks at 1644 and 1725 cm^1^ developed in samples after fermentation were pointed to the presence of a keto group (C=O) stretch bond, which was formed by CHO functioning on the cholesterol substrate. These results are supported by Saranya et al. [41], who used FTIR analysis to follow up or detect cholesterol metabolites formed throughout the breakdown process utilizing *Pseudomonas* sp., *Bacillus* sp. and *Streptomyces* sp. on cholesterol-containing medium. According to these findings, the C=O stretch bond and the ketone functional group may be seen in the frequency range of 1710–1665 cm^1^. According to the FTIR study, cholesterol degraded into the metabolites 4-cholestene-3-one, cholest- 4-ene-3, 17-dione, and androst-1, 4-diene-3, 17-Dione, these metabolites are cholesterol ketonic derivatives according to their chemical composition. After deterioration, the cholesterol’s functional groups deteriorated as well, resulting in ketones (C=O stretch bond). According to the results of the FTIR analysis, the primary metabolite produced might contain 4-cholestene-3-one. This speculation is supported by the findings of Wu et al., [42], who report the presence of C=O structure in FTIR with *V*_max_ at 1672.3 cm^−1^, attributed to the presence of 4-cholesten-3-one for matching the standard IR spectrum of 4-cholesten-3-one in the Sadtler Standard Infrared Grating Spectra (number 28840K) [43].

According to the difference in the DSC thermogram of the control sample and the fermented product, it was observed that the melting temperature peak showed at 99.26, heat flow − 3.6 mW/mg, heat capacity (− 924.69 mJ) and heat enthalpy (− 385.29 J/g), all of which were attributed to the action of CHO generated by *Streptomyces* sp. AN and the release of keto derivatives of cholesterol with distinct characteristics. At the end of the fermentation process, CHO acting on an existing cholesterol substrate caused a change in thermal characteristics. The melting temperature of the released ketonic derivatives of cholesterol was frequently lower than that of the original substrate.

## Conclusion

CHO, a member of the oxidoreductase family that catalyzes the oxidation of cholesterol molecules, has several applications. CHO has been thoroughly researched and utilized commercially for detecting the value of cholesterol in clinical samples, bioconversion of cholesterol into useful chemicals, food preparation and insecticidal efficacy against cotton weevils. Thus, the focus of this research has been directed toward the development and optimization of CHO, as well as the study of the enzyme’s mode of action using FTIR and DSC for its substrate before and after fermentation.

## Data Availability

All data generated or analysed during this study are included in this published article.

## Abbreviations

CHOs: Cholesterol oxidases
PB: Plackett-Burman
BB: Box-Behnken
RSM: Response surface method
FTIR: Fourier-transform infrared spectroscopy
DSC: Differential scanning calorimetry
SM: Selective medium
4-AAP: 4-Aminoantipyrine
PCM: Phase-contrast microscope
SEM: Scanning electron microscopy
CMC: Carboxymethyl cellulose
PCR: Polymerase chain reaction
NJ: Neighbour joining
MP: Maximum parsimony
LB: Luria-Bertani
S: Streptomyces
